# Functional traits associated with plant colonizing and competitive ability influence species abundance during secondary succession: Evidence from subalpine meadows of the Qinghai–Tibetan Plateau

**DOI:** 10.1002/ece3.4110

**Published:** 2018-06-01

**Authors:** Hui Zhang, Wei Qi, Kun Liu

**Affiliations:** ^1^ Institute of Tropical Agriculture and Forestry Hainan University Haikou China; ^2^ State Key Laboratory of Grassland Agro‐ecosystems School of Life Sciences Lanzhou University Lanzhou China

**Keywords:** functional traits, plant colonizing and competitive ability, species abundance, succession, trait–abundance relationship

## Abstract

It is widely recognized that colonists and competitors dominate early and late succession, respectively, with selected species having different colonizing and competitive abilities. However, it remains unknown whether colonizing and competitive ability can determine species abundance directly over succession. The data for five key functional traits were collected (photosynthesis rate, leaf turgor loss point, leaf proline content, seed mass, and seed germination rate), which are direct indicators of plant competitive and colonizing abilities including growth, drought and cold stress resistance, dispersal, and seed dormancy. Here, we tested the effects of colonizing and competitive abilities on species abundance, by employing a linear mixed‐effects model to examine the shifts in the relationship between species abundance and these five colonization and competition‐related traits in species‐rich subalpine secondary successional meadows (at 4, 6, 10, 13 years of age, and undisturbed, respectively) of the Qinghai–Tibetan Plateau. The abundant species at the early‐successional meadows tend to have high photosynthetic rate, high leaf proline content, low seed mass, and seed germination rate for having high colonizing ability, but low competitive ability. By contrast, late‐successional communities tend to be dominated by species with high competitive ability, but low colonizing ability, indicated by large seeds, high seed germination rate, low photosynthetic rate, and leaf proline content. The observed directional shifts in the relationships between traits (photosynthetic rate, leaf proline content, seed mass, and seed germination rate) and abundance with successional age, bring two new understandings of community assembly during succession of subalpine meadows in the Qinghai–Tibetan Plateau. First, it discloses that the differences in species abundance over succession can be directly attributed to differences in colonizing and competitive abilities of different species. Second, it expands the effects of multiple life historical differences including growth, resource competitive ability, cold stress resistance, dispersal, and seed germination strategy, represented by functional traits on community assembly along succession, that is, from the species to the community level.

## 1 INTRODUCTION

The factors that determine species abundance during ecological succession are among the oldest questions in community ecology (Bhaskar, Dawson, & Balvanera, 2014; Cornwell & Ackerly, 2010; Gleason, 1927; Shipley, Vile, & Garnier, 2006; Whittaker & Goodman, 1979). Succession begins with colonization; therefore, colonists (e.g., forb) possess high colonizing, but low resource competitive abilities, which dominate early succession (Marcante, Winkler, & Erschbamer, 2009; Matthews, 1992; Raffl & Erschbamer, 2004). As succession proceeds, however, resources (e.g., light, water, and soil nutrients) that affect plant growth and survival tend to decline. The initial colonists are progressively replaced with resource competitors (e.g., graminoid) that have high resource competitive ability, but low colonizing ability, and competitors therefore dominate late succession (Tilman, 1990). The shift in species dominator from colonist to competitors during succession is known to be maintained across different geographic regions and is consistent across floras, life forms, and phylogenetic groups (Cadotte, 2007; Cutler, Belyea, & Dugmore, 2008; Pulsford, Lindenmayer, & Driscoll, 2016; Tilman, 1990; Walker & Wardle, 2014). Colonist and competitor albeit possess different colonizing and competitive ability, and it remains unclear whether the differences in species abundance during succession can be directly attributed to differences in colonization and competitive ability of different species.

Plant functional traits are chosen to represent key aspects of species’ ecophysiology, morphology, and life history, and it can therefore be good indicators of plant colonizing and competitive abilities (Cadotte et al., 2006; Coomes & Grubb, 2003). For example, low seed mass ensures high dispersal ability, but lower resource competitive ability (Levine & Rees, 2002; Turnbull, Coomes, Hector, & Rees, 2004), whereas high seed mass confers stronger resource competitive ability, but may result in decreased dispersal (Moles & Westoby, 2004). Similarly, early colonizers are most likely fast‐growing species requiring a high photosynthetic rate; by contrast, resource competition would not favor fast‐growth strategies thereby having a low photosynthetic rate (Granath, Strengbom, & Rydin, 2010; Lohbeck et al., 2013; Zhu, Song, Li, & Ye, 2013). Therefore, testing the shifts in relationships between functional traits and abundance during succession, can facilitate uncovering whether the differences in species abundance can be directly attributed to differences in colonizing and competitive abilities of different species.

In this study, natural successional chronosequences of species‐rich subalpine meadow plant communities in the Qinghai–Tibetan Plateau were utilized to test whether differences in colonization and competitive abilities of different species can affect the differences in species abundance during succession. The successional communities range from those with no history of crop cultivation (undisturbed for at least 40 years), to those that have not been cultivated for 4 to 13 years. Three physiological traits (photosynthesis rate, leaf proline content, and leaf turgor loss point) and two reproductive traits (seed mass and seed germination rate) were selected to reflect plant colonizing and competitive abilities. The selection of the functional traits was based on the unique environmental conditions and the characteristics of the species at the study location**—**decreased nitrogen availability induced nitrogen competition, low annual precipitation (<600 mm), extremely cold temperature (the lowest daily temperature of 5°C) in the growing season (summer), and seed dispersal time (winter) (Liu et al., 2013; Zhang et al., 2015), which may select species with different colonizing and competitive ability. For instance, initial relatively high nitrogen availability will guarantee early colonist to have high colonizing ability (fast‐growing and high dispersal), but low nitrogen competitive ability, whereas nitrogen competition in late succession may force species to possess high nitrogen competitive ability, but low colonizing ability (slow‐growing and low dispersal) (Zhang et al., 2015). Typically, a high photosynthetic rate is linked to a rapid‐growth rate (Kirschbaum, 2011), while seed mass is considered to be a pivotal indicator of the trade‐off between colonization and competitive capacities (Coomes & Grubb, 2003). Hence, photosynthetic rate and seed mass are suitable for capturing plant colonizing (fast‐growing and high dispersal) and nitrogen competitive ability.

For plants, colonization is defined as survival through germination and establishment; however, colonization should include multiple life history strategies, (i.e., stress tolerance and different germination strategies), but not only fast‐growing and high seed dispersal (Cadotte et al., 2006). The low annual precipitation (<600 mm) and the extremely low temperature in the growing season may force colonist to have high drought and cold stress resistance for colonization. The leaf proline content and turgor loss point are direct measures of plant tolerance/resistance to abiotic stresses (Bartlett, Scoffoni, & Sack, 2012; Krasensky & Jonak, 2012); therefore, it is pertinent for testing the effects of drought and cold stress resistance on species abundance. In addition, the dispersal time (winter) and nitrogen competition in late succession may force species to have different germination strategies (Liu et al., 2013; Zhang et al., 2015). For instance, a low seed germination rate is an indicator of high seed physiological dormancy, which decreases postdispersal mortality of seeds that have been dispersed in the winter, when water availability is limited (Finch‐Savage & Leubner‐Metzger, 2006; Hu et al., 2013). However, the high nitrogen competitive environment in late succession may lead to late‐successional species having high rates of germination (i.e., accelerated germination) (Orrock & Christopher, 2010).

Specifically, it is expected to reveal whether the differences in species abundance during succession can be directly linked to differences in colonization and competitive abilities of different species, by testing the patterns of relationships between species abundance and these five competition and colonization‐related traits during succession. It is hypothesized that ample spatial availability for colonization, high nitrogen accessibility, drought and cold stress, and seed dispersal will encourage abundant species in early succession, with high photosynthetic rate, leaf proline content, small seeds, and a low seed germination rate, for having high colonizing ability including fast‐growing, high abiotic stress tolerance, high dispersal, and high seed dormancy, but low competitive ability. In contrast, due to nitrogen competition, late‐successional communities tend to be dominated by species with relatively large seeds and a high seed germination rate, low photosynthetic rate, and leaf proline content, which guarantees species to have high competitive ability, but low colonizing ability.

## 2 MATERIALS AND METHODS

### 2.1 Study sites

Sampling was conducted in the eastern portion of the Qinghai–Tibetan Plateau, Hezuo, China (34°55**′**N, 102°53**′**E), at a mean elevation of approximately 3000 m above sea level. This region is characterized by a cold and dry climate, with a mean annual temperature of 2.4°C, and mean annual precipitation of 530 mm, which was primarily distributed from July to August. The local vegetation in undisturbed sites is dominated by herbaceous species, such as *Elymus nutans* (Griseb), *Kobresia pygmaea* (C. B. Clarke), and *Thermopsis lanceolata* (R. Br) (Zhang et al., 2012). All sampled sites, except for those that were employed as control meadows (undisturbed for at least 40 years), had been utilized agriculturally to grow highland barley in the recent past with the farming being stopped from 4 to 13 years ago in different meadows. Here, we referred to the duration for which these meadows that had not been farmed for 4, 6, 10, and 13 years, as the “successional ages.”

### 2.2 Field sampling

Sampling was conducted in August (the peak growing season) of 2014 in meadows with five different successional ages (4, 6, 10, 13 years, and undisturbed for at least 40 years). Within a large landscape near Hezuo city, we identified two spatially distinct study areas with the same successional chronosequences for which farming histories might be reliably obtained by interviewing local farmers. These two areas (named chronosequence 1 and chronosequence 2) were ~10 km apart and had comparable topographic characteristics (e.g., orientation and slope). We therefore had two independent replicates for each successional age, yielding a total of 10 sites (successional meadows).

At each site, we randomly selected an area of 120 × 120 m^2^ and subsequently arranged 30 quadrats (0.5 × 0.5 m^2^) regularly in six parallel transects, with 20‐m intervals between adjacent quadrats. Species abundance was quantified as the total aboveground ramets of each species found within the 30 quadrats for each site.

### 2.3 Measurements of photosynthesis rate, leaf turgor loss point, and leaf proline content

At each site, the maximum photosynthesis rate of each species was measured between 9:00 a.m. and 12:00 a.m. during sunny days, with a portable photosynthesis system (Li‐6400, Li‐Cor, Lincoln, Nebraska, USA). Based on preliminary trials, the photosynthetic photon flux density was set at 1,500 μmol m^−2^ s^−1^ to ensure that light‐saturated photosynthetic rates were measured for all species (Zhang et al. unpublished data). Ambient CO_2_ and air temperature were maintained at 370 μmol mol^−1^ and 26°C, respectively. Five leaves from different individuals were selected per species for photosynthetic measurements.

Three to five plants were harvested during the early morning, sealed in black bags, and transferred to the laboratory. The plants were rehydrated until the leaf water potential exceeded −0.05 MPa, after which the leaves were detached for pressure–volume curve determination. Leaf weight and water potential were periodically measured during slow desiccation in the laboratory. Following all balanced pressure–weight measurements, the leaves were oven‐dried for 72 hr at 70°C to obtain their dry weight. The leaf turgor loss point was determined using a pressure–volume relationship analysis program, developed by Schulte and Hinckley (1985).

To measure the leaf proline content, we collected 20 leaves for each species. The second or third leaf from as many as 20 individuals was sampled from separate quadrats, if species abundance and frequency of occurrence permitted this. The proline content of leaves was measured as described by Marín, Andreu, Carrasco, and Arbeloa (2009), based on proline's reaction with ninhydrin.

### 2.4 Determination of seed mass and seed germination rate

Seeds were collected from meadows that were in close proximity to the sampling sites at the onset of their dispersal period (August to October). We travelled throughout the study areas on multiple occasions using different routes to collect seeds of as many species as possible to ensure that our database would represent the entire community. Seeds collected for a particular species were derived from more than 20 individual plants, except when the species was rare. Soon after collection, we cleaned and air‐dried the seeds, stored them dry, and allowed them to ripen at room temperature. For each species, three subsamples of 100 seeds were randomly selected and weighed to determine the average seed mass.

To determine the seed germination rate, we examined the seed viability of each species using the triphenyl tetrazolium chloride test (TTC) prior to seed germination experiments (Ruf & Brunner, 2003). The seed germination experiments were conducted in incubators (Conviron E15 Growth Chamber, Controlled Environments Ltd., Winnipeg, Canada) following the method of Liu et al. (2013). For each species, there were three replicates of 50 randomly selected seeds, which were incubated on filter paper moistened with distilled water in Petri dishes (9 cm in diameter) in darkness, with a relative humidity within the chambers of ~70%. The seeds were checked daily for germination, at which time they were exposed to light for several minutes. Thus, any light requirement by the seeds was likely fulfilled during these exposures (Baskin & Baskin, 1998). Germinated seeds (radicle visible) were removed from the Petri dishes at each counting, and water was added to the filter paper as required. To control for mold infections, we checked for germination and also checked for fungi. If any fungal infections were detected on the seeds, we rinsed them with distilled water and subsequently introduced them into a new Petri dish. The duration of the germination test was 60 days.

### 2.5 Statistical methods

As our study design involved two replicate meadow sites for each successional age, with species measurements undertaken within meadows of all ages, a hierarchical regression model was warranted (Gelman & Hill, 2006). The trait–abundance intercepts and slopes were subject to random effects, and our key objective here was to model intercepts and slopes of the trait–abundance relationship as a function of successional age, while allowing for possible differences in these relationships between the two chronosequences. We therefore used the linear mixed‐effects modeling framework and implemented the “lmer” function in the R package “lme4” (Bates, Maechler, Bolker, & Walker, 2014), to specify the linear regression model. Here, the intercept and trait–abundance slope were subject to random effects with “chronosequence” as a grouping factor (two factor levels) under which the successional age group “age.group” was nested (five factor levels). Successional age (in years) “age” was used as a meadow‐level predictor of trait values in one model. For each trait, we used the following model:

(1) Abundance=Age+Trait:Age+(1|chronosequence)

This model tests (1) whether abundance differs with age (age effect) by considering chronosequence as random effect and (2) whether slopes of abundance to trait differ with age, by considering chronosequence as random effect. Across all sites, both abundance and trait values were strongly skewed to right, and we thus log‐transformed both abundance and trait data to better meet the normality assumption and then performed both the linear mixed‐effects model.

## 3 RESULTS

**Figure 1:**
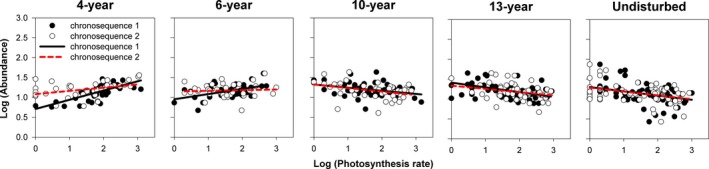
The relationships between species abundance and photosynthetic rate associated with successional age, while accounting for the random effect of chronosequence tested. Each point represents the mean value of a single species. Fitted lines are generated from linear mixed‐effects model (formula: abundance** **~ Age** **+ Trait:Age + (1|chronosequence)) with corresponding significance (*p*)

**Figure 2:**
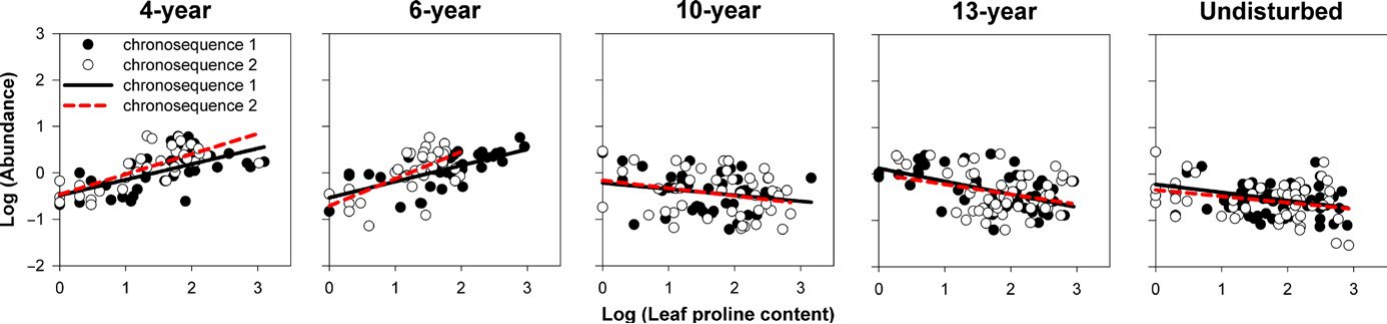
The relationships between species abundance and leaf proline content associated with successional age, while accounting for the random effect of chronosequence tested. Each point represents the mean value of a single species. Fitted lines are generated from linear mixed‐effects model (formula: abundance** **~ Age + Trait:Age + (1|chronosequence)) with corresponding significance (*p*)

**Figure 3:**
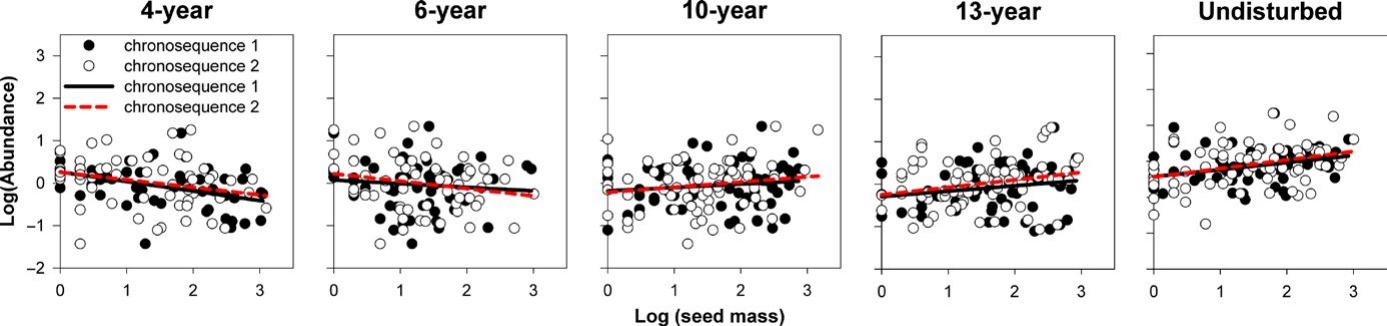
The relationships between species abundance and seed mass associated with successional age, while accounting for the random effect of chronosequence tested. Each point represents the mean value of a single species. Fitted lines are generated from linear mixed‐effects model (formula: abundance** **~ Age + Trait**:**Age + (1|chronosequence)) with corresponding significance (*p*)

**Figure 4:**
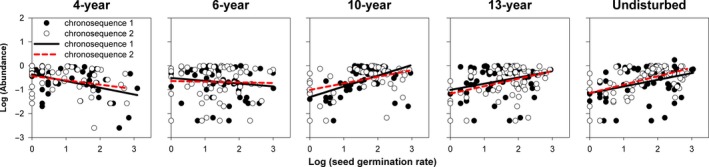
The relationships between species abundance and seed germination rate associated with successional age, while accounting for the random effect of chronosequence tested. Each point represents the mean value of a single species. Fitted lines are generated from linear mixed‐effects model (formula: abundance** **~ Age + Trait**:**Age + (1|chronosequence)) with corresponding significance (*p*)

The linear mixed‐effects model results indicated that the slopes of the relationships between species abundance and functional traits (photosynthetic rate, leaf proline content, and seed germination rate) changed significantly (*p* < .001) with successional age (Table 1). These results were evident in the changing slopes of trait–abundance relationships from the scatter plots and fitted regressions (Figures 1, 2, 3, 4). Photosynthetic rate and leaf proline content of plants were significantly positively correlated with species abundance in early‐successional (4‐ and 6‐year) meadows and negatively correlated in late‐successional (10‐, 13‐year, and undisturbed) meadows (Figures 1 and 2; *p* < .05). By contrast, seed germination rate was significantly negatively correlated with abundance in the 4‐year‐old meadows and positively correlated with abundance in the 10‐, 13‐year, and undisturbed meadows (Figures 3 and 4; *p* < .05).

**Table 1:** The relationships between species abundance and functional traits with successional age, while accounting for the random effect of chronosequence tested using the linear mixed‐effects model (formula: abundance ~ Age + Trait:Age + (1|chronosequence)). The degrees of freedom (*df*), *F*‐statistics, and corresponding *p*‐values are shown for the three functional traits. Boldface type indicates significance at *p* < .05

Source	*df/F/p*	Age	Trait:Age
Photosynthetic rate	*df*	4	5
	*F*	28.27	22.13
	*p*	**<.001**	**<.001**
Leaf proline content	*df*	4	5
	*F*	9.92	41.72
	*p*	**<.001**	**<.001**
Seed mass	*df*	4	5
	*F*	12.61	25.72
	*p*	**.01**	**<.001**
Seed germination rate	*df*	4	5
	*F*	14.9	15.11
	*p*	**<.001**	**<.001**
Leaf turgor loss point	*df*	4	5
	*F*	0.13	0.58
	*p*	.97	.72

For leaf turgor loss point, the linear mixed‐effects model result indicated a nonsignificant (*P* = .93) change in the trait–abundance relationship with successional age (Table 1, Figure S1). Leaf turgor loss point was not significantly associated with species abundance in any of the successional meadow communities (Figure S1; *p* > .05).

## 4 DISCUSSION

Using replicated chronosequences of species‐rich subalpine meadow communities in the Qinghai–Tibetan Plateau, we observed directional shifts in the relationships between species abundance and traits (photosynthesis rate, leaf proline content, seed mass, and seed germination rate) during succession. As it was hypothesized, ample area for colonization, high nitrogen availability, cold stress, and special seed dispersal time (winter) in the early‐successional stages allowed abundant species with high colonizing ability including fast‐growing, high abiotic stress tolerance, high dispersal, and high seed dormancy, but low competitive ability indicated by a high photosynthetic rate, leaf proline content, small seeds, and a low seed germination rate to thrive. In contrast, resource competition resulted in late‐successional communities that were dominated by species with relatively large seeds and a high seed germination rate, low photosynthetic rate, and leaf proline content, which guarantees species to have high competitive ability, but low colonizing ability.

Previous studies at this site have shown consistent floristic changes during succession in these meadow communities. Early‐successional communities tend to be dominated by life forms such as forbs that are annual plants and good colonist, whereas older meadows are increasingly dominated by graminoids that are mostly perennial and good resource competitors (Liu et al., 2013; Zhang et al., 2015). Thus, colonists (forbs) such as *Geranium phlzowianum* and *Potentilla ansrina* are abundant across early stages at both sites, while competitors (graminoids) dominate at later stages include *Elymus nutans Griseb* and *Kobresia pygmaea (C. B. Clarke*). Corresponding differences in colonizing and competitive ability represented by plant functional traits are expected from early to late succession, due to the shift in dominator from colonist to competitor, but have not been linked directly to species abundance (Zhang et al., 2015). Moreover, the shift in plant strategies from colonization to competition has since been originated from models that allow the degree of competitive asymmetry and preemption in a community to occur on a continuum (Calcagno, Mouquet, Jarne, & David, 2006; Figueiredo & Connolly, 2012) and the responding empirical evidence for linkage between species abundance and such a shift remains extremely sparse, especially in natural ecosystems (Pastore et al., 2014). The observed directional shifts in the relationships between colonization/competition‐related traits (photosynthesis rate, leaf proline content, seed mass, and seed germination rate) and species abundance with successional age provided strong empirical evidence for the influence of the shift in plant strategies from colonization to competition on species abundance along a naturally developed subalpine meadow succession.

A number of studies have demonstrated that shifts in light capture‐related traits (e.g., photosynthesis rate and SLA) and reproduction traits (e.g., seed mass) during succession are attributed to light availability induced a trade‐off in plant growth strategies, wherein fast‐growing species with high photosynthetic rates, but low reproduction traits, characterize species that occur in the high resource (e.g., light) environments of early‐successional stages, while the increasing resource limitation of later successional stages selects species that can persist at low resource levels but with low photosynthesis rate, but high reproduction traits. However, these studies have generally focused on the effects of light availabilities on successional changes in functional traits at individual species, but not at the community level (Cornwell & Ackerly, 2010). The observed directional shifts in the relationships between life history traits (photosynthesis rate and reproduction traits (seed mass and seed germination rate)) and species abundance with successional age expand the effects of plant multiple life historical differences including growth, resource competitive ability, dispersal, and seed germination strategy on community assembly over successions, that is, from the species to the community level.

Liu et al. (2013) found significant differences in daily temperature at different successional meadows during the growing season, with the lowest daily temperature of 5°C near the soil surface in early‐successional meadows and 10°C near the soil surface in late‐successional meadows. They also found that the daily temperature range was ~5–25°C near the soil surface in early‐successional meadows and ~10–20°C near the soil surface under vegetation in late‐successional meadows. Although it remains to be investigated whether these temperature differences reflect general patterns across early‐ and late‐successional meadows in the landscape, it suggests that early‐successional species experience relatively greater cold stress than late‐successional species and may, therefore, possess greater cold stress resistance. As leaf proline content is highly correlated with cold stress resistance, abundant species in early species may have high leaf proline content for having high cold stress resistance. In addition, Sanchez et al. (2002) found that nitrogen deficiency may cause proline degradation, thereby reducing leaf proline content. Indeed, we have found in an earlier study that nitrogen content in late‐successional meadows was lower than that in early‐successional meadows (Zhang et al., 2015). Therefore, the observed shift from a positive slope (in 4‐ and 6‐year‐old meadows) to a negative slope (in 10‐, 13‐year‐old, and undisturbed meadows) in the relationship between leaf proline content and species abundance might be initiated by cold stress coupled with nitrogen deficiency.

It should be noted that, given the generally low annual precipitation at the study site, moisture limitation may be expected to be of importance; thereby, affecting species abundance over succession (Bartlett et al., 2012). However, there were no observed significant changes in leaf turgor loss point–species abundance relationships along the successional gradient. Although the total annual precipitation in this region is low (occurring primarily during the growing season, from July to August), the soil appears to be water saturated (Zhang, Gilbert, Wang, Liu, & Zhou, 2013). This may explain the nonsignificant correlations between leaf turgor loss point and species abundance, suggesting that moisture limitation may not be critical for plant growth and survival during the growing season in our study system. In addition to the concentrated precipitation during the growing season, the generally cool summers likely resulted in negligible evaporation from the soil, which might contribute to the sufficient amount of moisture in the soil.

Overall, the observed directional shifts in the relationships between four colonization/competition‐related traits (photosynthesis rate, leaf proline content, seed mass, and seed germination rate) and species abundance with successional age led to two new understanding of community assembly of secondary succession in these subalpine meadows. First, differences in species abundance during succession can be directly attributed to differences in colonizing and competitive abilities of different species. Second, the significant trait–abundance relationships expanded the effects of functional traits on community assembly during succession, that is, from the species to the community level. Nevertheless, our data have limitations, as we include only 10 study sites although from two independent successional chronosequences, which might limit the power of our analyses. Moreover, the study focused on aboveground functional traits, yet belowground traits representing specific root functions could be important in determining species abundance, especially in habitats with limited nutrients and/or moisture, such as the subalpine meadows.

## CONFLICT OF INTEREST

None declared.

## AUTHOR CONTRIBUTIONS

H.Z. designed research; W.Q., K.L., and H.Z. performed research; H.Z. analyzed data; H.Z. wrote the manuscript.
