# Supplementary material for: Functional traits associated with plant colonizing and competitive ability influence species abundance during secondary succession: Evidence from subalpine meadows of the Qinghai–Tibetan Plateau
Source: Ecol Evol. 2018 Jun 1;8(13):6529–36. doi: 10.1002/ece3.4110 (PMC6053576; doi:10.1002/ece3.4110)

### Fig. S1 The relationships between species abundance and leaf turgor loss point associated with successional age, while accounting for the random effect of chronosequence tested. Each point represents the mean value of a single species. Fitted lines are generated from linear mixed effect model (formula: abundance ~ Trait + Age + Trait : Age + (1|chronosequence)) with corresponding significance (P).


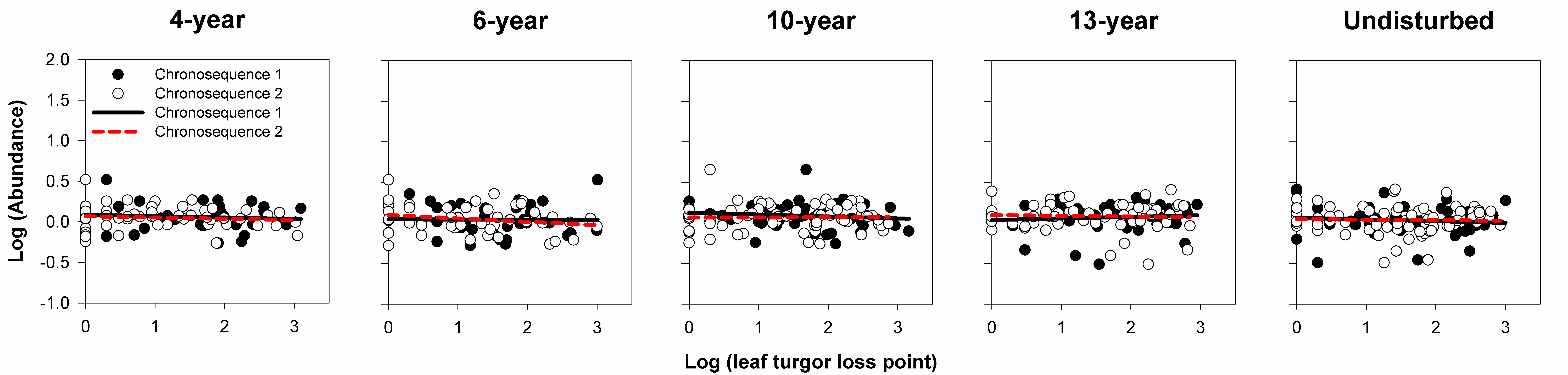


**Fig. S2** The scatter plot of species abundance and photosynthetic rate associated with successional age. Each point represents the mean value of a single species.


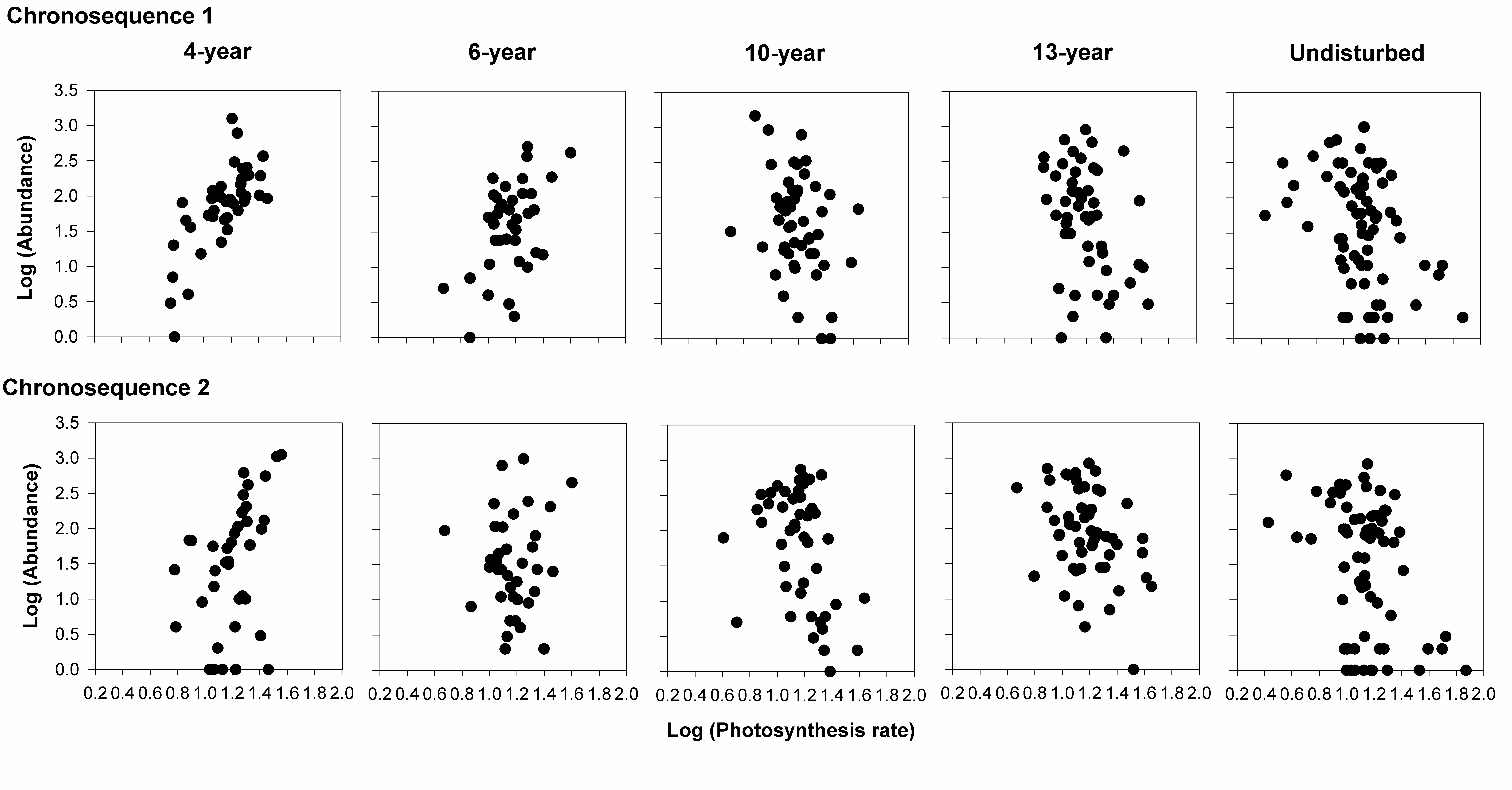


**Fig. S3** The scatter plot of species abundance and leaf proline content associated with successional age. Each point represents the mean value of a single species.


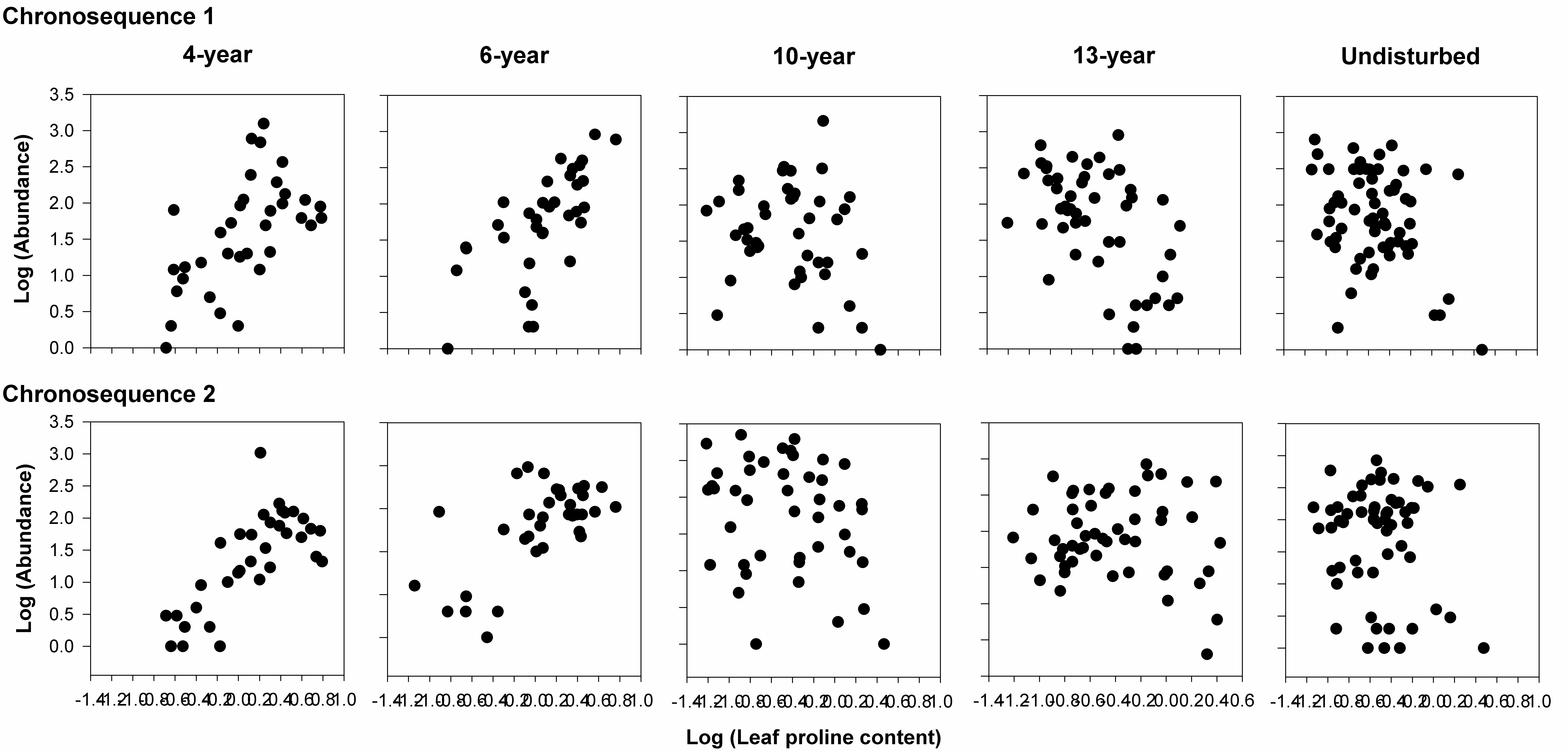


**Fig. S4** The scatter plot of species abundance and seed mass associated with successional age. Each point represents the mean value of a single species.
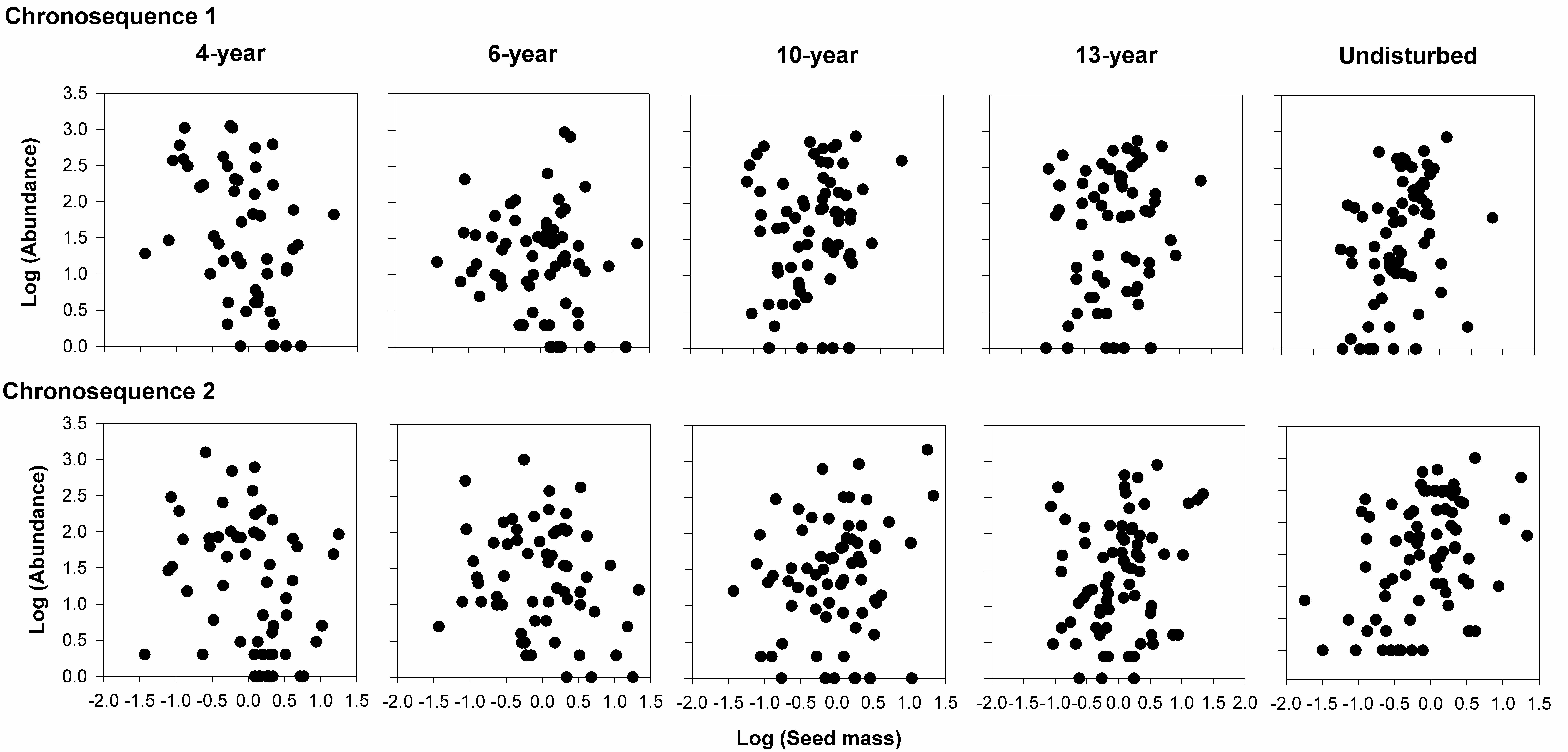


**Fig. S5** The scatter plot of species abundance and seed germination rate associated with successional age. Each point represents the mean value of a single species.


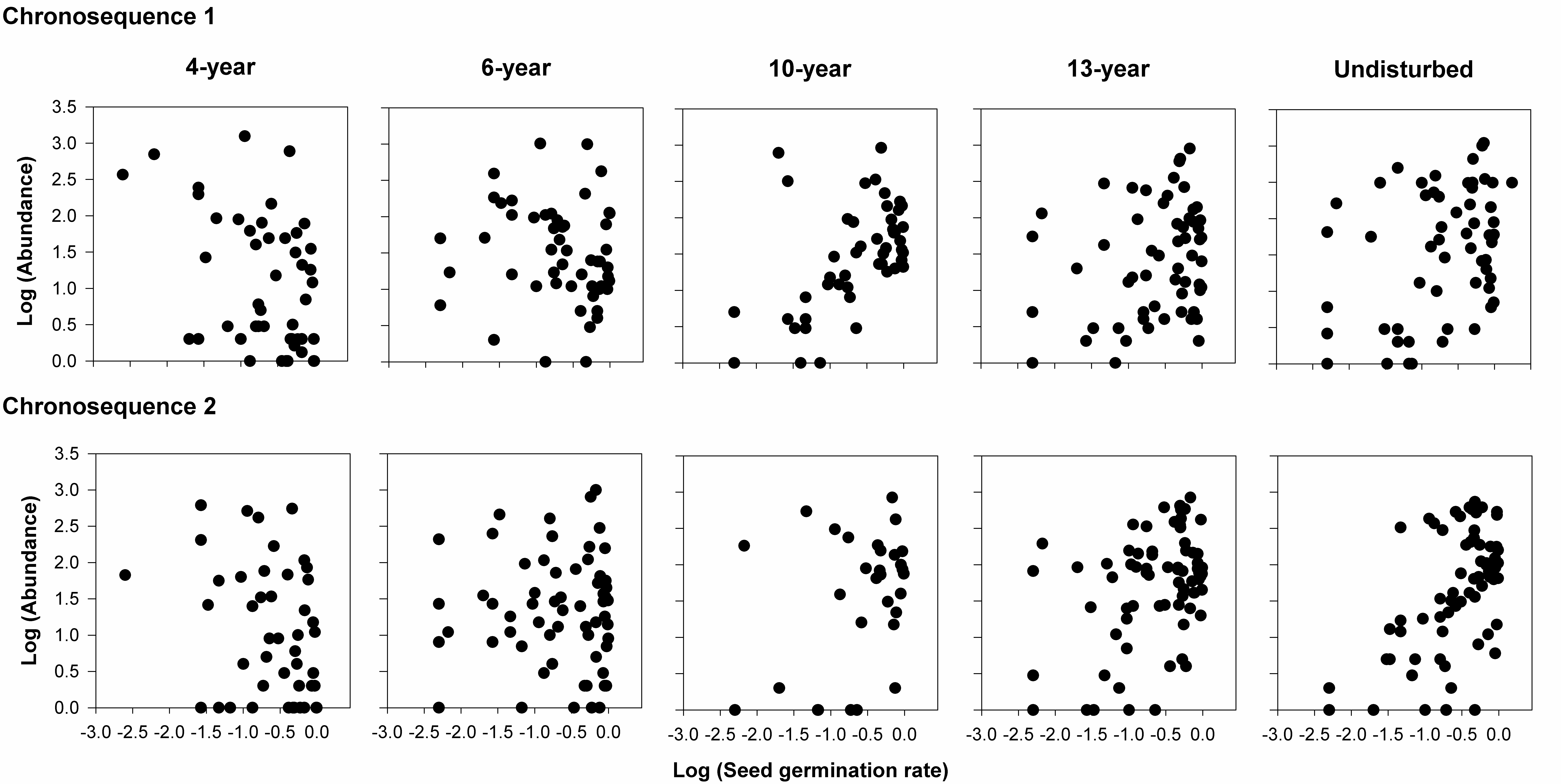


**Fig. S6** The scatter plot of species abundance and leaf turgor loss point associated with successional age. Each point represents the mean value of a single species


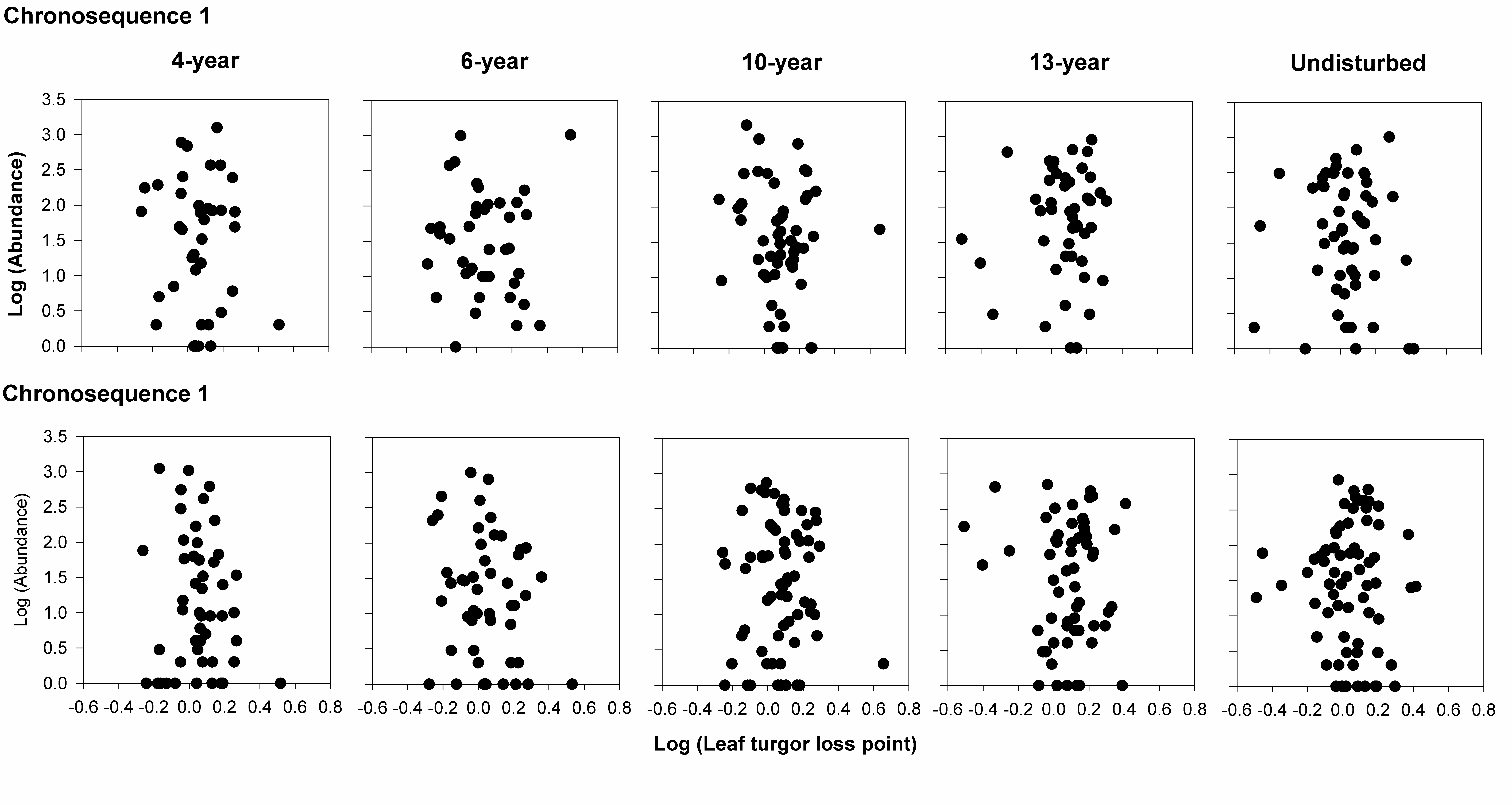

Supplement: Supplementary file 1 [file ECE3-8-6529-s001.doc]
